# Exploring cellulose nanocrystals obtained from olive tree wastes as sustainable crop protection tool against bacterial diseases

**DOI:** 10.1038/s41598-022-10225-9

**Published:** 2022-04-12

**Authors:** Daniele Schiavi, Sara Francesconi, Anna Rita Taddei, Elena Fortunati, Giorgio M. Balestra

**Affiliations:** 1 Department of Agriculture and Forest Sciences (DAFNE), University of Tuscia, Via S. Camillo de Lellis Snc, 01100 Viterbo, Italy; 2 High Equipment Centre, Section of Electron Microscopy, University of Tuscia, Largo dell’Università Snc, Blocco D, 01100 Viterbo, Italy

**Keywords:** Biotic, Bacterial development, Antiparasitic agents, Microbial ecology, Microbe

## Abstract

Nanomaterials in agriculture represent one of the most innovative method for protecting crops, due to possibility of being applied as nanopesticides or nanocarriers for active ingredients. Furthermore, nanotechnology could be combined with the concept of circular economy through the opportunity of obtaining highly technological nanometric materials starting from agro-industrial wastes. The present research evaluated the possibility of synthesizing cellulose nanocrystals (CNCs) from olive pruning wastes through chemical bleaching, reusing them as sustainable tool to control the causal agent of the olive knot disease (*Pseudomonas savastanoi* pv. *savastanoi*). CNCs showed an interesting in vitro effect in inhibiting bacterial growth and bacterial biofilm formation, as well as the ability of reducing bacterial epiphytic survival in a comparable way to copper sulphate on leaf surfaces, when used at 1% w/v. CNCs were at the same time investigated for their interaction with olive tree seedlings, showing no negative effects on leaf development, and a promising root uptake, indicating that CNCs could be used also as nanocarriers for active ingredients. Obtained results highlight the innovative possibility of designing sustainable plant protection strategies capable of revalorise lignocellulosic wastes, meaning a simultaneous low environmental impact thanks to reduction of traditional agrochemicals input.

## Introduction

Olive tree (*Olea europea* L.) belonging to the *Oleaceae* is an evergreen woody plant with shrubby and low-pitched habitus. Olive tree domesticated in the Mediterranean area, leading to its cultivation to extract the oils contained in the drupes^1^. In Italy, olive tree cultivation is mostly concentrated in the South, especially in the Apulia Region, comprising more than 1.7 million hectares^2^. Italy is the second olive oil world producer, after Spain, counting around 400,000 tons of olive oil produced per year. The Italian olive germplasm is extremely variegated since more than 200 cultivars exist; among them, the traditionally most employed ones for cultivation are Frantoio, Leccino, Dritta, Coratina, Cerasuola, and Bosana^3,4^. In the recent years, the olive tree cultivation has been negatively impacted by climate change and by the spread of pathogens and pests^5^. Among them, *Pseudomonas savastanoi* pv. *savastanoi* (ex Smith 1908) Gardan et al., 1992 (Psav) is a Gram-negative bacterium causal agent of the olive knot disease^6^. Psav has an epiphytic phase living on the external surfaces of the plant, such as leaves, branches, and shoots. Nevertheless, the bacterium reaches a high population density in spring and autumn, when olive trees are most susceptible^7^. At humid conditions, the pathogen penetrates the plant woody tissues through microlesions and spread in the parenchyma. At this point, the bacterium synthesises auxins and cytokinins leading to the formation of hyperplastic and hypertrophic tissues, the tubercles, mostly present on the branches and, rarely, on fruits and roots. After the rupture of the tubercle, Psav will disseminate and provoke further infections^8,9^. The management of the olive knot is based on appropriate agronomical practices, such as the employment of balanced pruning allowing the circulation of air inside the canopy, and on preventive chemicals, such as cupric salts, applied in post-harvest, after pruning or unfavourable atmospheric events, such as hailstorm^10,11^.

Cupric salts represent in fact fundamental pesticides for management of phytopathogenic bacteria in conventional and organic agriculture, preventing and reducing infection, avoiding of bacterial cells penetration into living plant tissues and/or reducing transmission of the disease from plant to plant in the course of the diffusion stage^12,13^. During the last 30 years, many studies have made it clear that cupric salts accumulate in soil, produce several phytotoxic effects and are harmful to humans^14,15^. In this regard, the EU Commission moved to reduce the amount of exploitable copper in control strategies, both in terms of field-usable quantities and formulations (Regulation EU 2018/1981 of 13 December 2018). Thus, pathogens control strategies based on low environmental impact and substitution of copper compounds are urgently required. Many researchers are focusing on the development of new eco-sustainable crop protection strategies for the management of bacterial plant diseases based on nanomaterials^16–18^. Nevertheless, there is still a relatively low percentage of naturally derived pesticides compared to the number of agrochemicals derived from chemical synthesis^19^. Effective pathogen management is a major challenge in modern agriculture, where control efficacy, cost affordability, environmental safety, toxicity towards non-target organisms, and sustainability of the production system are important factors^20^. In this regard, the use of plant-derived molecules appears extremely promising. Several researches were developed in the last years since the apparent possibility of using lignocellulosic nanomaterials in agriculture as eco-friendly nanocarriers. Lignin, hemicellulose and cellulose-based nanocompounds were proposed for different agri-food applications, from plant nutrition to post-harvest protection, mostly due to their low-cost synthesis, biocompatibility and abundancy, but also considering the recent developments in biopolymers manipulations. Encapsulation and surface modification are the most promising strategies for exploiting lignocellulosic nanocarriers in plant protection, since a wide range of active molecules were reported to be successfully tested for controlling plant pests and pathogens^21^. However, there is still a big lack in explaining and studying the antimicrobial properties of lignocellulosic nanomaterials when used alone.

Among the enormous variety of plant-derived molecules, cellulose is the most frequent found on Earth. Cellulose is a crystallin biopolymer composed of glucose and constitutes the plant cell wall^22^. Cellulose is a solid, non-toxic, colourless, and odourless polymer possessing high mechanical strength and absorption capacity, biocompatibility, and hydrophilicity. Due to its unique properties, cellulose-based polymers have been investigated for potential applications in plant protection, since agro-food wastes can be valorised to extract cellulose by employing low-impact methods in a context of circularity^23^. Indeed, cellulose can be easily hydrolysed in cellulose nanocrystals (CNCs), characterized by a diameter of 1–100 nm and a length of 50–500 nm^24^, showing interesting properties, such as low density and coefficient of thermal expansion and high elasticity. Furthermore, CNCs are non-toxic, biocompatible, and display several hydroxyl groups allowing chemical modifications on the surface; thus, CNCs are promising nanocarriers delivering active antimicrobial molecules^25,26^. Several research studies already demonstrated that CNCs-based agrochemicals can be employed as diseases management strategies^27–30^. As an example, a recent research demonstrated CNCs-based agrochemicals to be highly effective for the management of *P. syringae* pv. *tomato* (Pst)*,* causing the bacterial speck on tomato plants. CNCs-based agrochemicals reduced Pst epiphytic survival acting as mechanical barrier on tomato leaves counteracting the pathogen ingress in plant tissues. Importantly, no phytotoxic effects were observed on tomato leaves after the application of CNCs-based agrochemicals^31^. Similar results were obtained in terms of reduced incidence and biocompatibility in hazelnut plants affected by bacterial blight when treated with CNCs extracted from lignocellulosic waste^32^.

For such evidences, nanotechnologies are extremely promising in order to ensure low-impact crop protection strategies by valorising plant-derived biomass wastes as a source of nanomaterials, thus favouring the affirmation of circular supply chains as much as the employment of alternative and eco-sustainable compounds as plant protection strategies, capable to support crop production and safeguard human health and ecosystems at the same time^33^. Considering such advantages, the aim of the present research work was to reuse and valorise olive tree pruning wastes for the extraction of cellulose and characterization of CNCs. After that, CNCs have been in vitro and in vivo assayed for their antibacterial activity against Psav and their biocompatibility on olive tree plants.

## Results

### Optimization of cellulose extraction from olive tree pruning wastes and synthesis of CNCs

Three different concentrations of sodium chlorite have been tested to optimise the bleaching phase in cellulose extraction process from olive pruning residues. One bleaching at 1% did not accurately separate the lignocellulosic components, thus CNCs synthesis was not possible. On the other hand, bleaching at 3% and 5% allowed the separation of lignocellulosic components and the cellulose yield obtained with the two protocols was statistically similar (31.8% and 34.3%, respectively, of cellulose yield compared to the starting pruning wastes biomass). Acidic hydrolysis was able to obtain CNCs from extracted cellulose, showing yield of 21.5% and 20.4% from the protocol comprising the bleaching at 3% and 5%, respectively, and, in both cases, the CNCs final concentration in the aqueous solution was of 0.29% w/v (Table 1).

**Table 1:** Cellulose yield (%), hydrolysis yield (%), CNCs concentration (%), Average CNCs length and width (nm), Cristallinity index for the three cellulose extraction protocols differing for the sodium chlorite concentration (1, 3, 5% w/v) employed for the bleaching step.

NaClO_2_ bleaching (% w/v)	Cellulose yield (%)	Hydrolysis yield (%)	CNCs concentration (% w/v)	Average CNCs lenght (nm)	Average CNCs width (nm)	Cristallinity index (%)
1	–	–	–	–	–	–
3	31.8 ± 1.0 a	21.5 ± 0.8 a	0.29 ± 0.02 a	81 ± 2 a	10 ± 0.4 a	64
5	34.3 ± 2.0 a	20.4 ± 1.6 a	0.29 ± 0.01 a	93 ± 4 a	10 ± 0.3 a	65

“–” means that was not possible to obtain pure cellulose, thus CNCs were not synthesised from the lignocellulosic matrix obtain by using bleaching at 1%. No statistical differences were recorded. “± value” refers to standard deviation between three replicates. All the data come from the average of three independent replicates.

### Characterization of cellulose-based derivates

**Figure 1:**
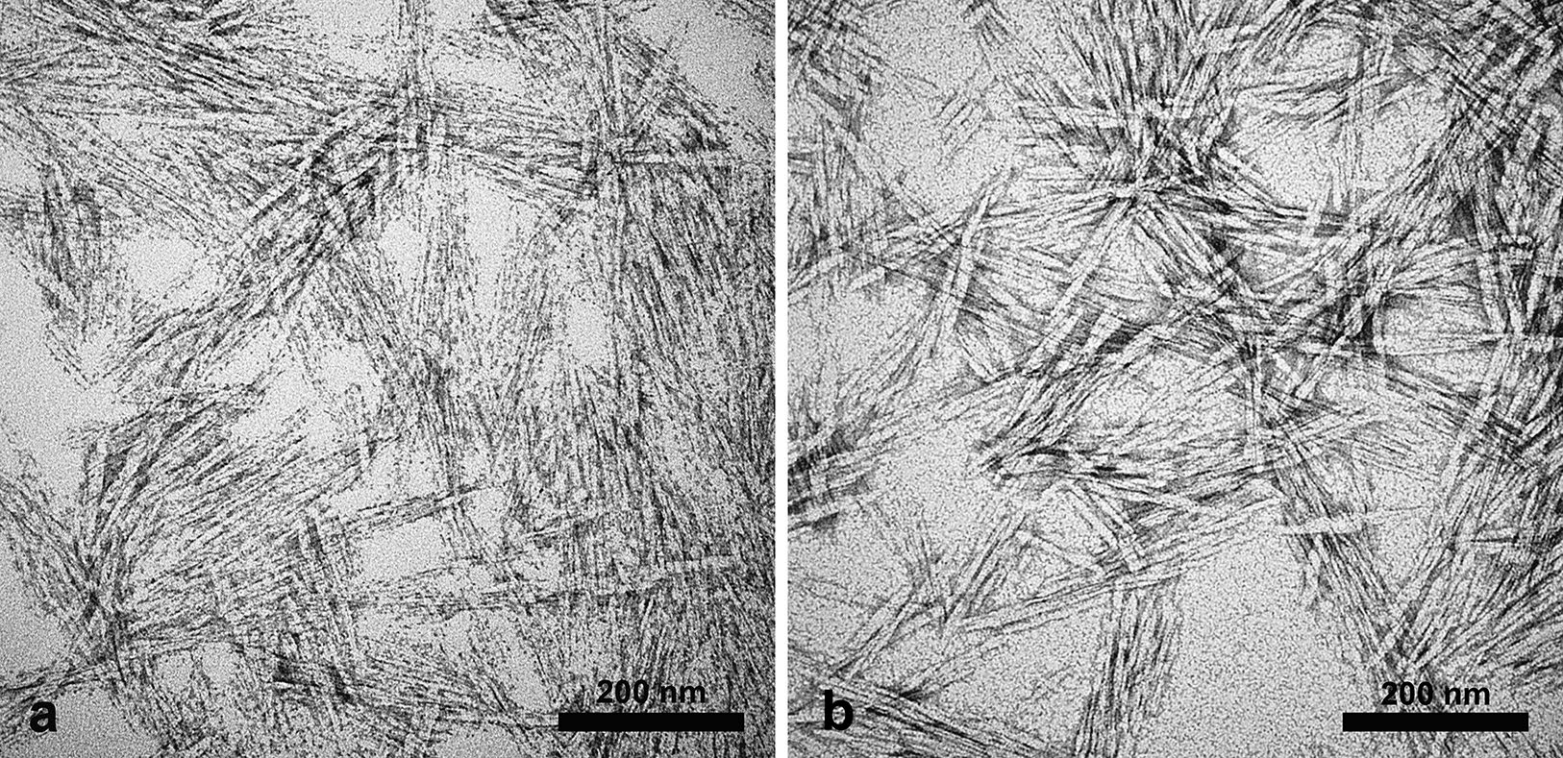
Ultrastructural characterization by TEM of CNCs bleached at 3% (**a**) and 5% (**b**).

TEM analysis on negatively stained CNCs evidenced needle-like and rod-shaped structures (Fig. 1) and their length was of 81 nm and 93 nm from bleaching at 3% and 5% respectively, while average width was 10 nm for both (Table 1). XRD analysis confirmed the crystalline nature of CNCs showing the presence of type I and type II cellulose, whilst the resulted crystallinity index was o 64% and 65% for cellulose obtained from bleaching at 3% and 5%, respectively. Since no statistical differences were observed in terms of morphology, chemical characteristics, and synthesis yield, CNCs obtained from extraction process with 3% w/v bleaching were used in all other experiments.

### In vitro antibacterial activity of CNCs

**Figure 2:**
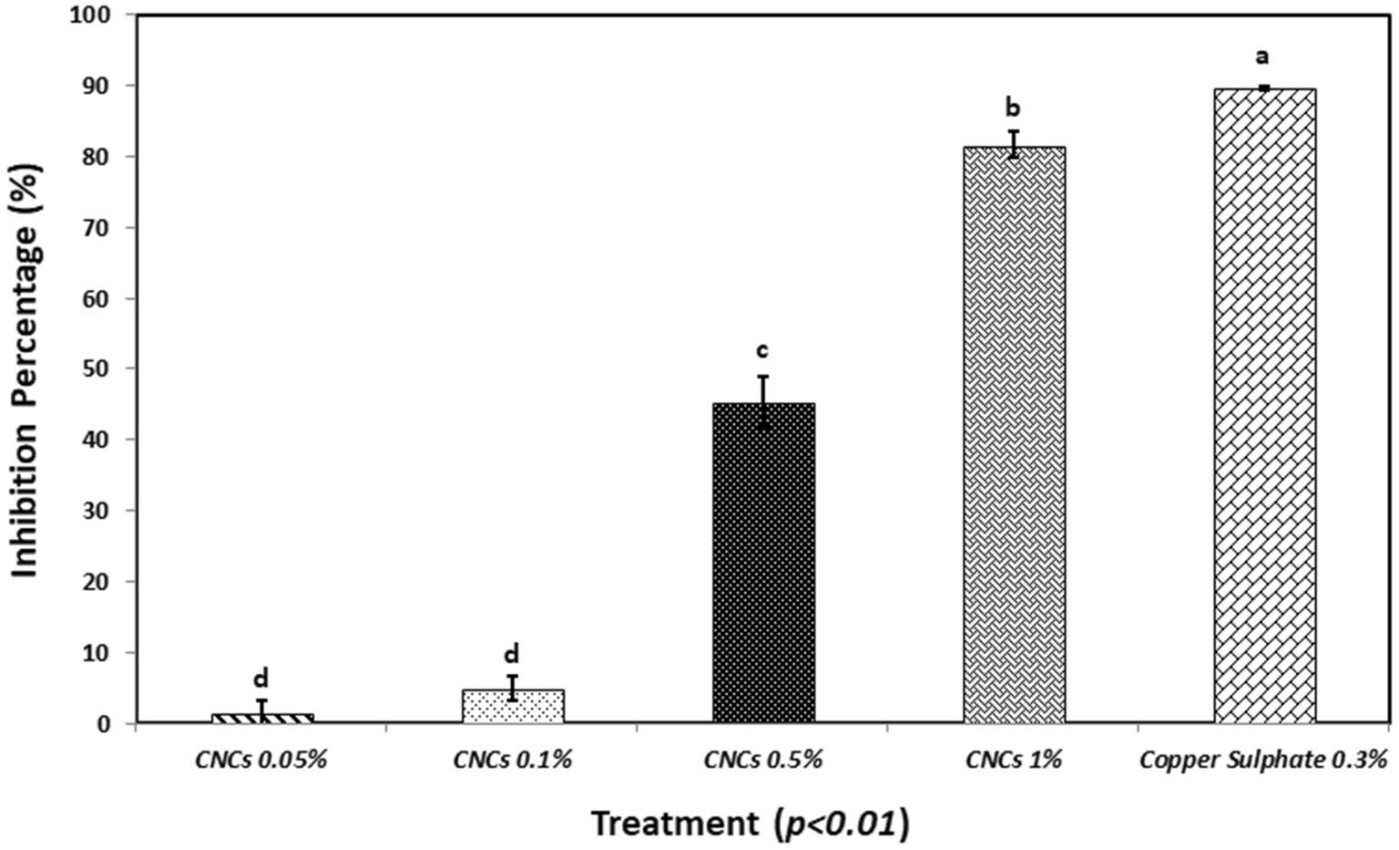
In vitro inhibition of *Pseudomonas savastanoi* pv. *savastanoi* by different cellulose nanocrystals concentrations (0.05, 0.1, 0.5, 1% w/v) at 48 h. Copper sulphate pentahydrate at 0.3% w/v was used as control. Statistical significance was based on a *p*-value < 0.01. Error bars represent the standard deviation between eight replicates. In vitro experiments were conducted thrice, and the effect of the experiment was not significant.

The microtiter assay revealed a high statistical significance in term of antibacterial activity against Psav, since the % of inhibition after 48 h was 1.3, 4.9, 45.2 and 81.6% respectively for treatments at 0.05, 0.1, 0.5 and 1% w/v of CNCs. Copper sulphate pentahydrate at 0.3% displayed a nearly total (89.7%) bacterial inhibition (Fig. 2).

**Figure 3:**
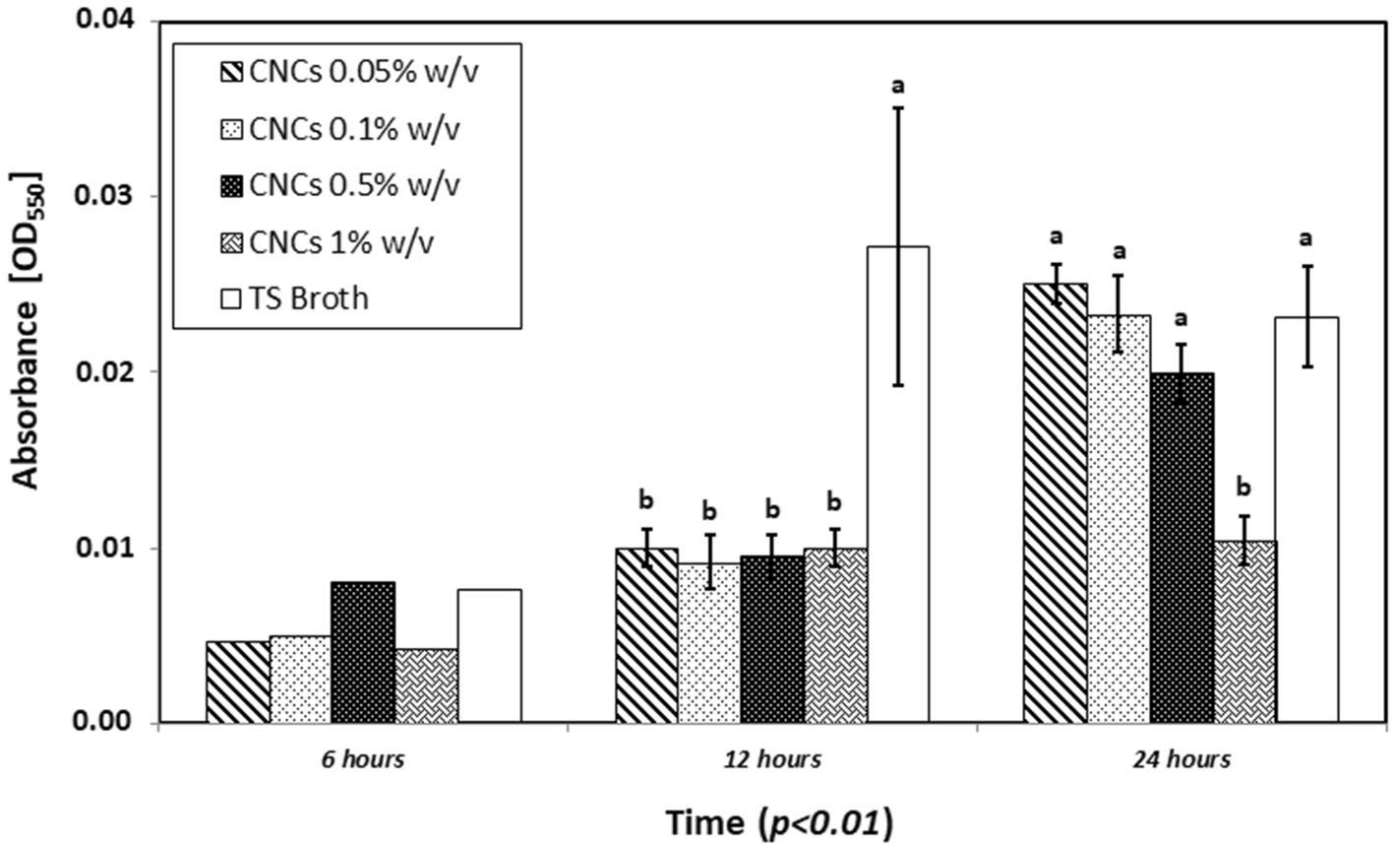
In vitro biofilm formation of *Pseudomonas savastanoi* pv. *savastanoi* by different cellulose nanocrystals concentrations (0.05, 0.1, 0.5, 1% w/v) at 6, 12 and 24 h. Tryptone soy broth (TS Broth) was used as control. Statistical significance was based on a *p*-value < 0.01. Error bars represent the standard deviation between eight replicates. In vitro experiments were conducted thrice, and the effect of the experiment was not significant.

The in vitro assay on bacterial biofilm at different times showed some potential effects of CNCs in counteracting the formation of Psav biofilm. While at 6 h from the inoculation no statistical differences between treatments could be appreciated, at 12 h all tested concentrations of CNCs showed a high statistical difference in comparison with the control (Tryptone Soy Broth). At 24 h only the highest tested concentration of CNCs (1% w/v) with a value of absorbance at 550 nm of 0.010 kept its ability in reducing bacterial biofilm concentration compared to the control (0.023 absorbance value at 550 nm) (Fig. 3). Since CNCs showed the most promising antimicrobial effects when used at 1% w/v, the same concentration was used in the next experiments.

### CNCs uptake in olive plants

**Figure 4:**
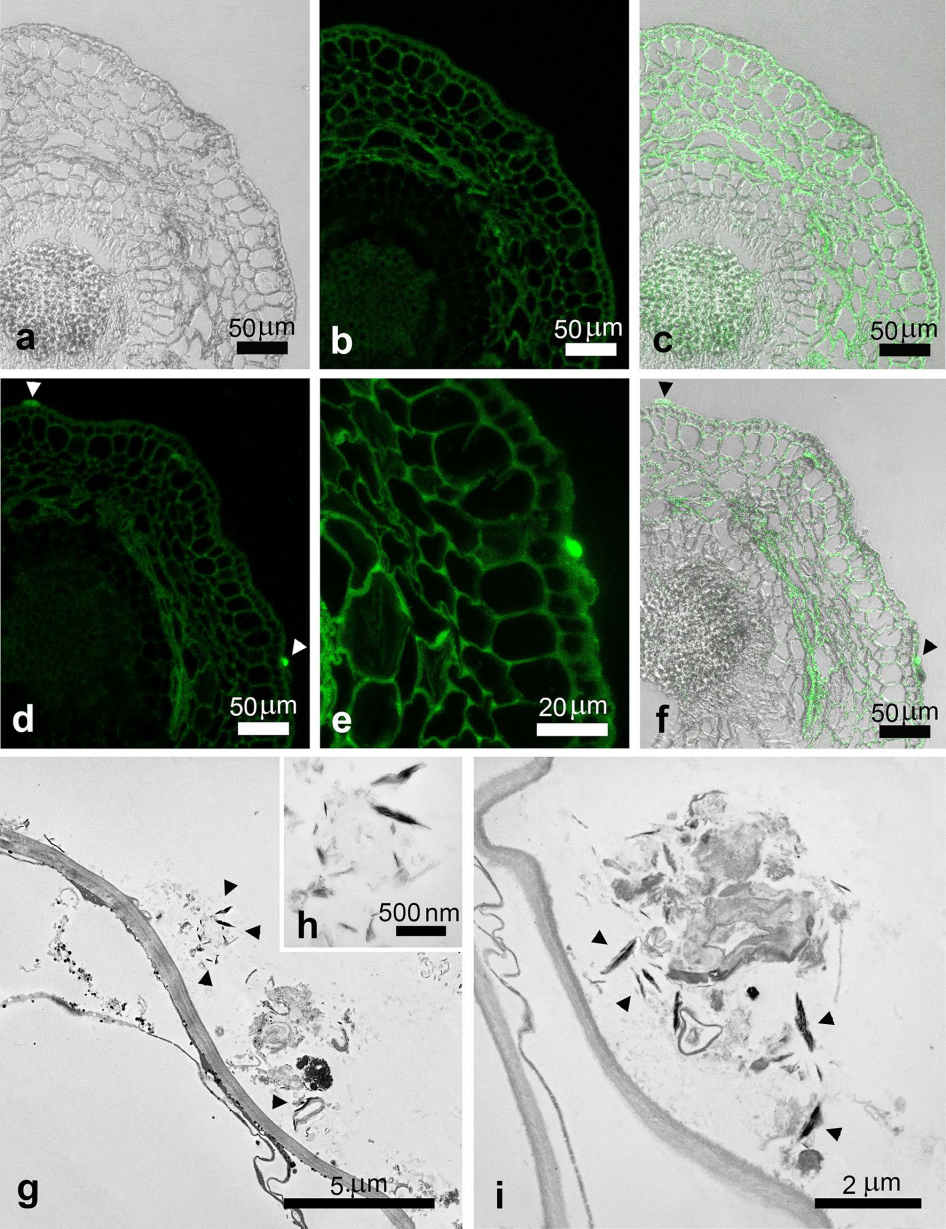
Differential Interference Contrast (DIC), confocal and merged-channels micrographs of control olive root in cross sections (**a–c**), where autofluorescence of cellulose was evidenced. In samples treated with 1% w/v CNCs some fluorescent spots were detected on the outer surface of the root ((**d–f**), arrowheads). Ultrastructural analysis by TEM evidenced the presence of needle-like electron dense structures attributable to CNCs, often associated with externally present amorphous material ((**g–I**), arrowheads).

**Figure 5:**
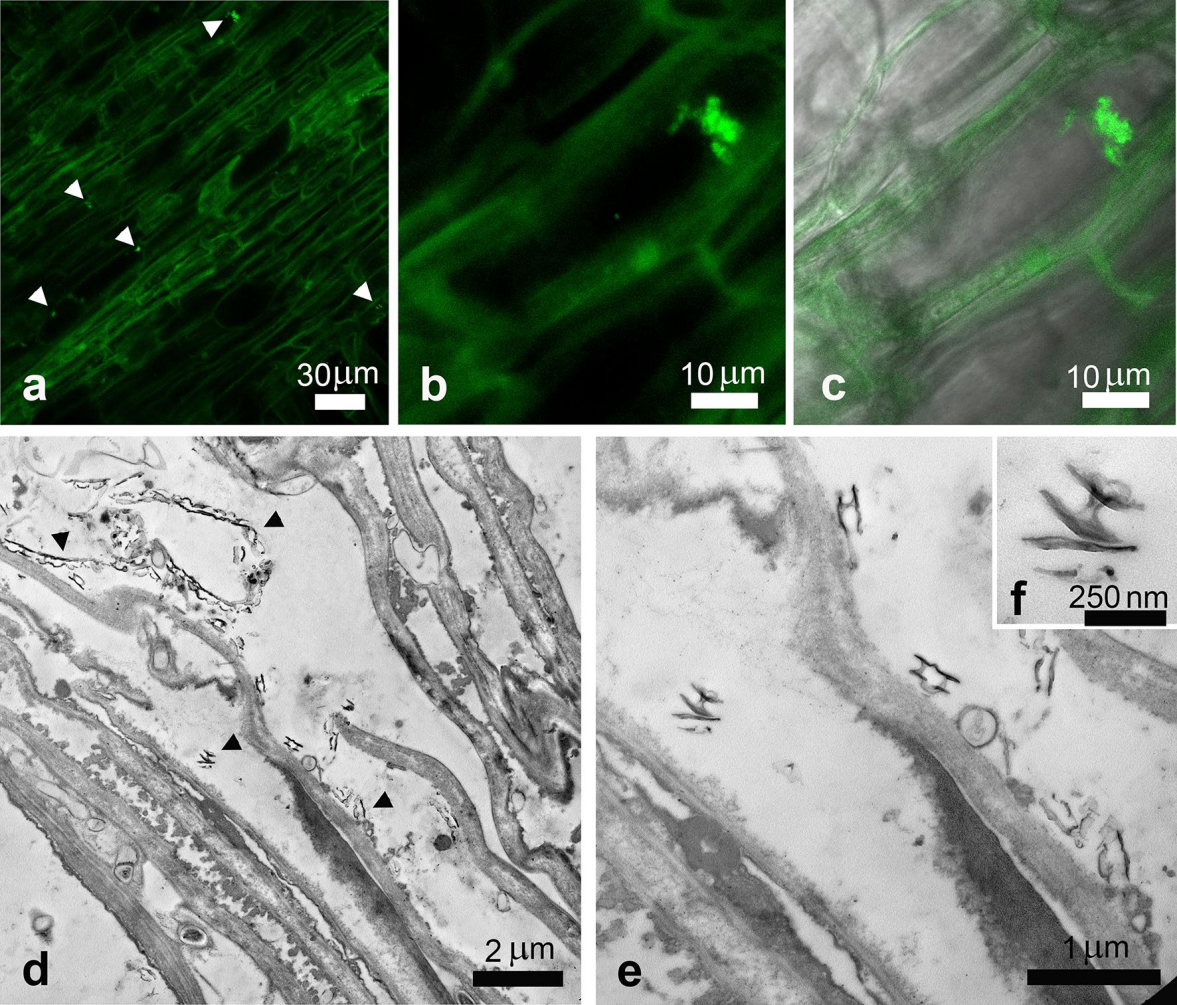
Confocal images of olive root treated with 1% w/v CNCs in longitudinal sections, where some fluorescent spots were revealed in the first layers of root parenchymatic cells ((**a–c**), arrowheads). Ultrastructural analysis by TEM evidenced the presence of needle-like structures in the cellular symplast, compatible with CNCs ((**d–f**), arrowheads).

To investigate the potential uptake of CNCs by plants, samples of olive roots, after being in aqueous suspension made with Hoagland solution and CNCs at 1% w/v for 48 h, were observed through confocal and transmission electron microscope. Thanks to the autofluorescence of cellulose, confocal observations revealed the presence of fluorescent clusters on the outside of roots, in contact with the cell wall, indicating, as confirmed by TEM, that CNCs aggregated on the root surface (Fig. 4). More notably the presence of fluorescent spots in the first layers of root parenchymatic cells were observed in treated samples, pointing out the presence of CNCs inside the plants. TEM observations corroborated the results, since the same electron dense acicular structures compatible with the dimensions of CNCs described before and observed outside the roots, were recognized in the cellular symplast (Fig. 5).

### In vivo biocompatibility and antibacterial activity of CNCs

**Figure 6:**
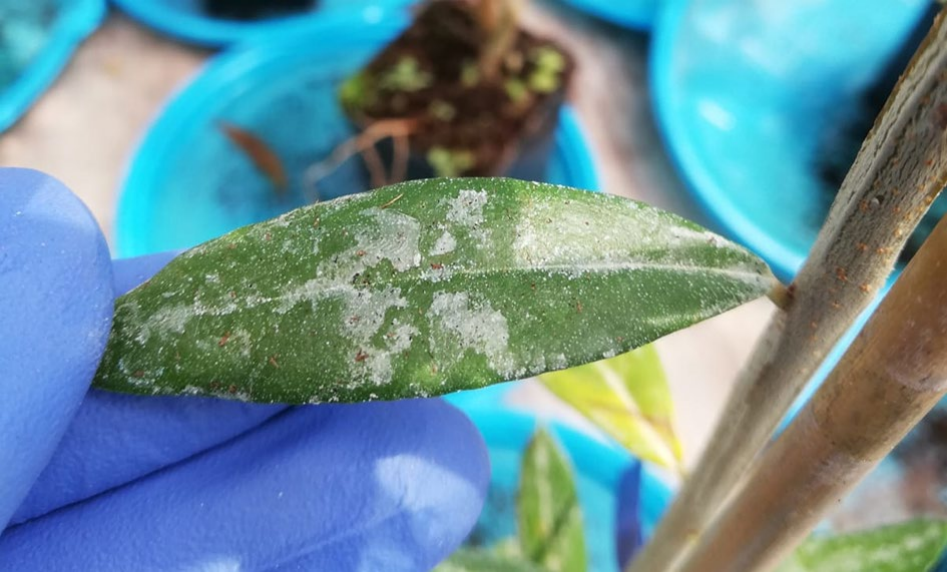
Observed patina on leaves treated with 1% w/v CNCs suspension.

CNCs biocompatibility has been evaluated by monitoring the Nitrogen Balance Index (NBI) and leaf surface area development after foliar treatment on olive tree seedlings. No statistically significant differences were recorded among NBI values recorded at 1, 7, 14 days post treatment (dpt). At the same time, also the leaf surface area values revealed no statistically significant differences among the treatments (Table 2). These results revealed that the application of CNCs did not display any phytotoxic effect on basal biological parameters of olive tree seedlings. Moreover, phenotypical observations highlighted the formation of a white film on the leaves surface of plant treated with CNCs at 1% (Fig. 6).

**Table 2:** Biological parameters of olive tree seedling treated with a CNCs suspension at 1% w/v, water and copper sulphate at 0.3% w/v measured at 1, 7 and 14 days after treatments.

Treatment	Biological parameters	Days post treatment		
		1 dpt	7 dpt	14 dpt
Water	Leaf area (cm^2^)	5.1 ± 0.3 a	4.9 ± 0.3 a	4.9 ± 0.5 a
	Chlorophyll content (DU)	51.9 ± 2.4 a	57.2 ± 2.4 a	56.3 ± 2.1 a
	Flavonols content (DU)	1.85 ± 0.07 a	1.94 ± 0.06 a	1.97 ± 0.06 a
	Nitrogen balance index	28.3 ± 1.5 a	29.9 ± 1.9 a	29.1 ± 1.8 a
Copper sulphate 0.3% w/v	Leaf area (cm^2^)	5.4 ± 0.4 a	5.0 ± 0.2 a	5.1 ± 0.3 a
	Chlorophyll content (DU)	50.3 ± 2.8 a	57.3 ± 1.4 a	53.6 ± 1.7 a
	Flavonols content (DU)	1.93 ± 0.05 a	1.91 ± 0.04 a	1.88 ± 0.05 a
	Nitrogen balance index	26.4 ± 1.9 a	30.1 ± 1.1 a	28.8 ± 1.4 a
CNCs 1% w/v	Leaf area (cm^2^)	5.3 ± 0.3 a	4.9 ± 0.4 a	5.1 ± 0.3 a
	Chlorophyll content (DU)	54.7 ± 2.4 a	50.7 ± 2.3 a	50.3 ± 2.0 a
	Flavonols content (DU)	2.01 ± 0.03 a	1.99 ± 0.05 a	1.97 ± 0.06 a
	Nitrogen balance index	27.4 ± 1.6 a	25.7 ± 1.6 a	27.4 ± 1.7 a

No statistical differences were recorded. “± value” refers to standard deviation between 20 measurements collected on ten plants per thesis. In vivo experiments were conducted thrice, and the effect of the experiment was not significant. Leaf area is expressed as square centimetres (cm^2^) while chlorophyll and flavonols contents are reported as Dualex Unities (DU).

**Figure 7:**
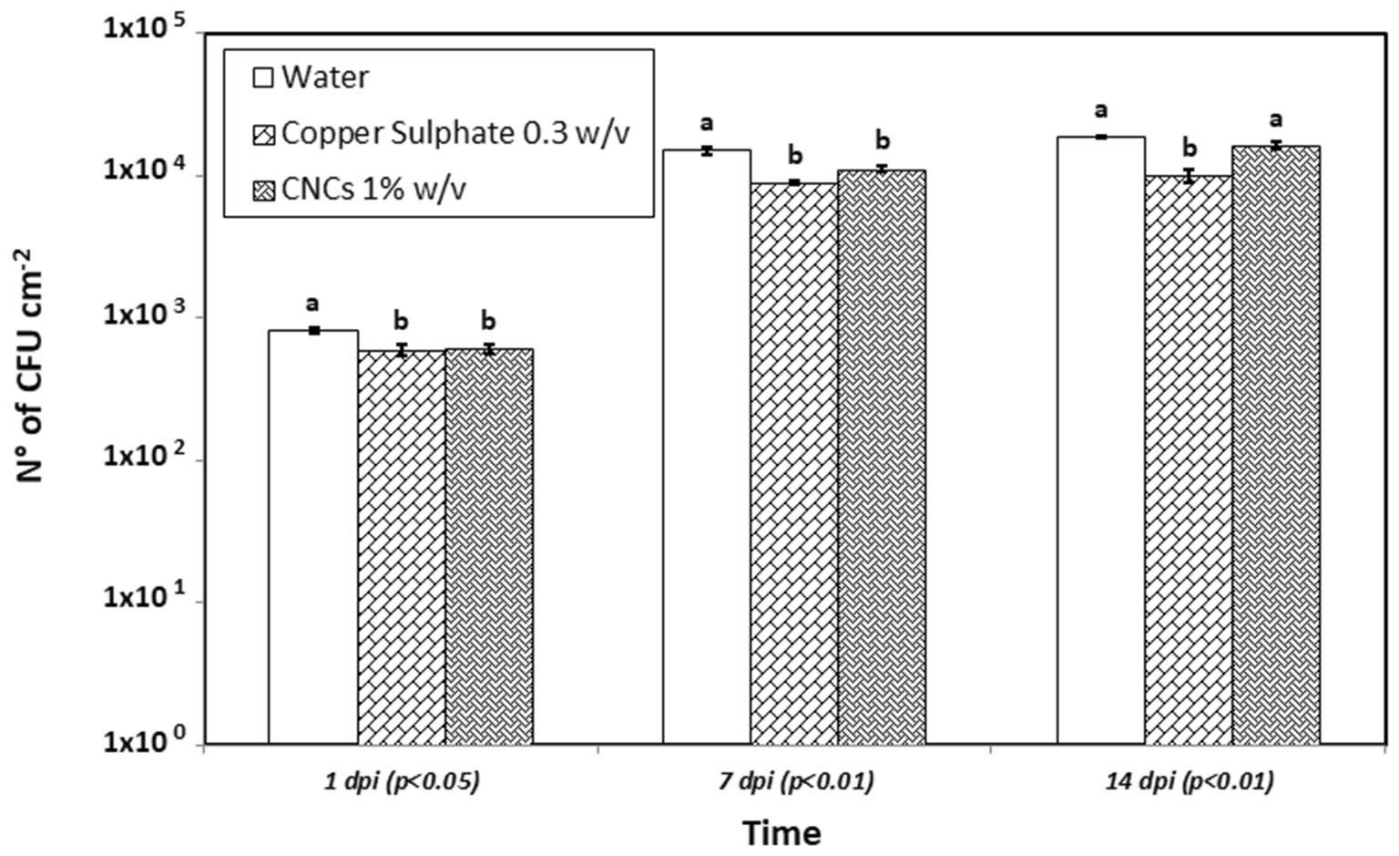
Epiphytic survival of *Pseudomonas savastanoi* pv. *savastanoi* in olive tree seedling treated with a CNCs suspension at 1% w/v, water, and copper sulphate at 0.3% w/v measured at 1, 7 and 14 days after inoculation. Statistical significance was based on *p*-value < 0.05 and *p*-value < 0.01. Error bars represent the standard deviation between three replicates. Each replicate consisted in one leaf collected from each seedling (ten leaves per thesis). In vivo experiments were conducted thrice, and the effect of the experiment was not significant.

In vivo antibacterial experiments revealed that at 1 day post inoculation (dpi), CNCs at 1% w/v reduced Psav epiphytic survival as much as copper sulphate pentahydrate at 0.3% w/v (6.1 × 10^2^ CFU/cm^2^ and 5.95 × 10^2^ CFU/cm^2^, respectively), while in water-treated plants, Psav epiphytic survival was of 8.11 × 10^2^ CFU/cm^2^. At 7 dpi, CNCs-based treatment decreased Psav inoculum as much as copper sulphate pentahydrate (1.11 × 10^4^ CFU/cm^2^ and 8.83 × 10^3^ CFU/cm^2^, respectively), while in water-treated plants, Psav epiphytic survival was of 1.49 × 10^3^ CFU/cm^2^. On the other hand, at 14 dpi CNCs did not display antibacterial activity, since their effect was comparable to the water treatment (1.62 × 10^4^ CFU/cm^2^ and 1.87 × 10^4^ CFU/cm^2^, respectively), while in copper sulphate pentahydrate treated plants, Psav epiphytic survival was of 9.92 × 10^3^ CFU/cm^2^ (Fig. 7).

**Figure 8:**
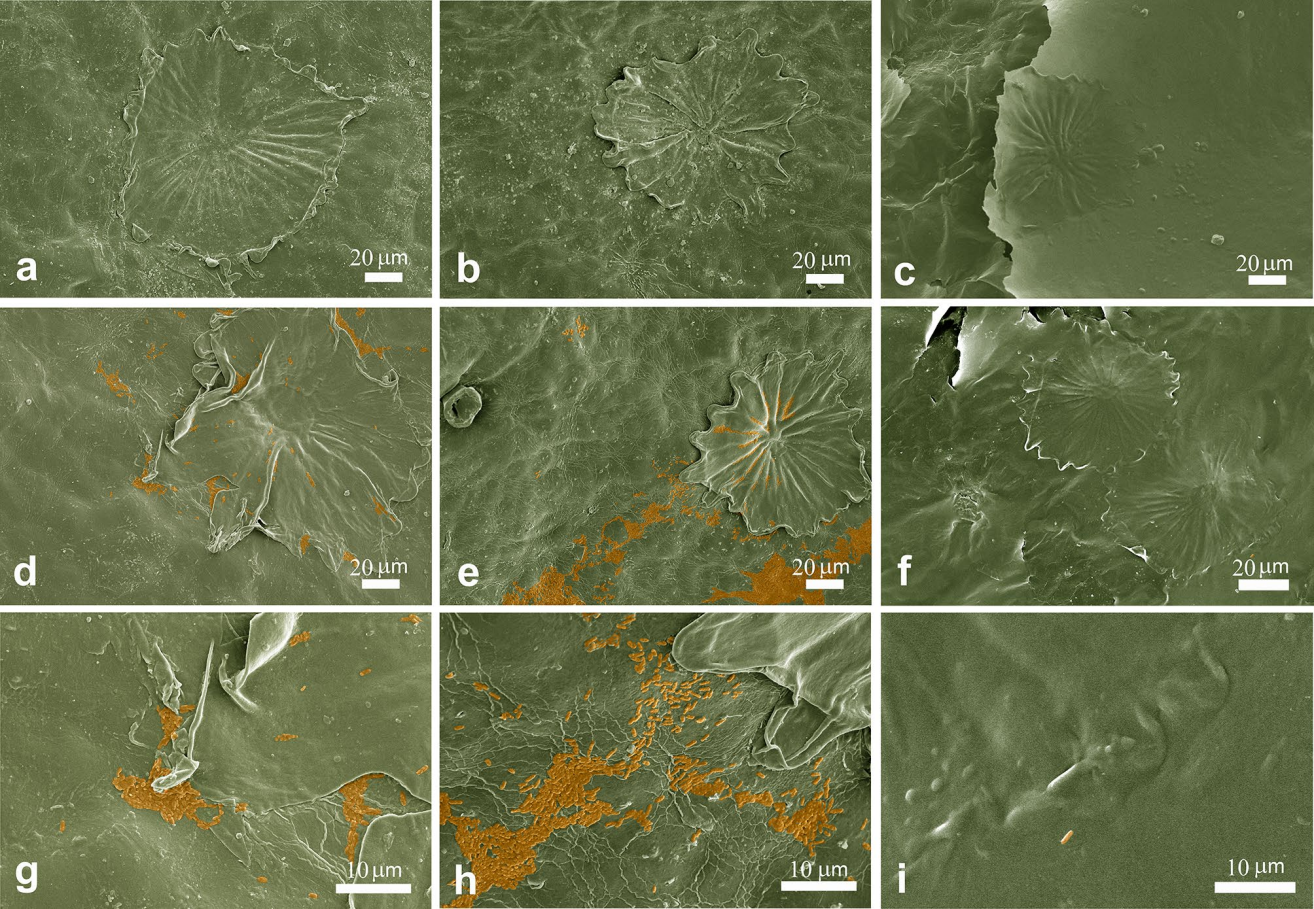
SEM analysis of control samples of olive leaves treated respectively with water (**a**), copper sulphate at 0.3% w/v (**b**) and CNCs suspension at 1% w/v (**c**) before the inoculation. Leaf surface showed the characteristic trichomes that were clearly distinguishable in (**a,b**), whereas were partially hided in (**c**) for the presence of the CNCs film covering the surface (**c**). After Psav inoculation of samples treated respectively with water (**d**), copper sulphate at 0.3% w/v (**e**) and CNCs suspension at 1% w/v (**f**), Psav colonies were visible as coloured areas on the leaves surface, very often located near the trichomes (1 dpi). The presence of bacteria is to be noted as abundant in (**d,e**), while only rare bacteria have been detected on the surface of the leaves treated with CNCs (**f**). Higher magnification of the previous images (**g–i**). Images were false-colourized.

SEM observation of leaf samples taken at 1 dpi (Fig. 8) evidenced Psav colonies on the leaf surface when water or copper sulphate at 0.3% w/v were used for the treatment, with bacteria often located near the trichomes. In leaves treated with 1% w/v CNCs, bacterial cells can hardly be detected. The patina observed in the in vivo experiment resulted as a superficial crust that homogenously enveloped the leaf upper surface and its trichomes.

## Discussion

### Extraction of cellulose, synthesis, and characterization of CNCs from olive tree pruning wastes

Olive tree pruning residues are some of the most abundant, renewable and cheap lignocellulosic residues generated in the Mediterranean countries, while representing an average estimated production of 3000 kg/ha per year worldwide^34^. Usually, olive tree pruning is eliminated to keep fields clean and prevent propagation of plant diseases by burning, grinding or scattering the biomasses on fields, causing economical costs and environmental concerns^35^. However, considering its large yearly production, and its sugars content, this lignocellulosic material has been proposed as a source for a broad range of high-value-added products^36^. Among such products, cellulose is the most abundant organic substance in olive tree pruning and it is an almost inexhaustible raw material. Cellulose can be considered as a renewable and biodegradable source of energy and raw material for other compounds’ production, possessing an easily modified chemical structure, which can produce a wide range of fibers, films and functional polymers^37^. Cellulose nanocrystals possess high aspect ratio, strength and thermal stability, low density, allow easy surface functionalization, high optical transparency, high water retention, and a large specific surface area which make this material suitable for different advanced applications, such as drug delivery^38,39^. In the present work, we successfully extracted cellulose and synthetized CNCs by olive tree pruning. Applied protocols indicated the possibility of obtaining cellulose and highly crystalline nanometric cellulose with an average length of 81 nm and an average width of 10 nm, by using a 3% w/v sodium chlorite bleaching followed by a 64% v/v sulphuric acid hydrolysis with a final yield of 21.5%. These results perfectly fit with the ones reported in previous studies, where CNCs extracted from several agricultural wastes were characterized. The average dimensions of biomass-based CNCs are often 10–15 nm for width and 100–150 nm for length^32,40,41^. In this work the acicular structure of CNCs was observed in both the samples obtained with different bleaching concentrations, although a smaller size in terms of length was measured in comparison with similar researches where nanocrystalline cellulose from olive residues was synthetized^42^. This difference could be explained considering the preliminary process for isolating cellulose from the lignocellulosic starting biomass: the more effective is the process of cellulose extraction, in terms of lignin and hemicellulose-free final fraction, that can be achieved by following treatments of sodium chloride and hydroxide at the proposed concentrations, the more effective is the acid hydrolysis in reducing cellulose fibers to their nanosized forms^43,44^. The studies indicated that is possible to lower the amount of used chemicals in extraction process without compromising the quality of CNCs. The XRD analysis revealed the presence of both I and II cellulose types and at the same time no differences in the crystal organization of obtained CNCs from both bleaching protocols. Cellulose I is known to be composed by celluloses Iα and Iβ, whit a more relevant presence of the latter in higher plants. Crystal structure of cellulose II is more stable than cellulose I, and it is produced as the result of the alkaline treatments performed with sodium hydroxide. XRD patterns showed in the crystalline final products the peculiar domains of cellulose II, even if the CNCs obtained from 5% w/v sodium chlorite peaks were slightly lower. This behaviour was already observed in other works where nanocrystalline cellulose was obtained from lignocellulosic biomasses^41^. Results pointed out the remarkable CNCs concentration obtained (almost 30% w/v) with a high crystallinity index (65%). Obtained results are moreover compatible with ones carried out by previous researches applied on lignocellulosic wastes for the isolation of cellulose and its nanoforms^40^.

### In vitro and in vivo antibacterial properties of CNCs against Psav

The focus of our research was to evaluate the potential application of CNCs from olive pruning wastes for an innovative management of Psav, in a context of circular economy. In vitro experiments displayed a dose dependent antibacterial activity for CNCs, reaching values of growth inhibition up to 80% when used at 1% w/v. Moreover, CNCs suspension were able to diminish the bacterial biofilm formation with a persistent effect for 24 h when used at the highest concentration. Promising results were noticed also in terms of Psav epiphytic survival in artificial inoculated olive tree seedlings. CNCs at 1% proved capable of maintaining low levels of epiphytic population on leaves up to seven days after the inoculum, in a comparable way to the ones showed by copper sulphate at the field dose. As far we know, these are the first results describing the potential in vivo antimicrobial activity of CNCs on a bacterial plant pathogen. Obtained results are indeed compatible with the ones showed in other works, where the influence of different nanocellulose forms were investigated on human bacterial pathogens. One of the mechanisms that could explain the antibacterial activity is related to the role as mechanical obstacle represented by CNCs to bacterial swimming in liquid media: the ability of TEMPO-mediated oxidized cellulose nanofibrils (CNFs) of limiting bacterial swimming was positively assayed on several foodborne pathogens (*E. coli*, *L. monocytogenes*, *B. aureus*, *S. typhimurium*) with inhibition values ranging from 8 to 60%. In the same study plates treated with CNFs were able to diminish the bacterial biofilm formation of *L. monocytogenes*^45^. Similar result were reported on *E. coli*, in terms of antibacterial activity and inactivation of bacterial cells in CNCs-coated surface^46^. Research’s authors proved that damage to bacterial cells is not provoked by oxidation phenomena, while assaying that a physical interaction between crystalline-shaped nanocellulose and bacterial membranes could be the principal stress mechanism explaining CNCs toxicity. On bacterial cells treated with CNCs it was possible to observe losses of membranes integrity due to the disruption of phospholipid bilayer, which is the main component in cellular membranes, confirmed on artificial lipid vesicles. The same approach positively confirmed the ability of lowering bacterial growth and biofilm formation on *P. aeruginosa* in comparison with a commercial wound dressing^47^. Furthermore, in this research it was proved that CNCs does not represent a viable carbon source for bacterial cells development. Another CNCs mode of action have been described by Sun et al.^48,49^, which investigated the effect of the application of CNCs on *P. aeruginosa* and *P. fluorescens* aggregation and adhesion. The studies demonstrated that an aqueous solution of CNCs induce bacterial flocculation, thus reducing the ability of bacterial cells to adhere to a solid surface through a depletion mechanism. Exclusion of CNCs in depletion zones next to bacterial cell surfaces maximizes the entropy of the system and drives the coagulation of bacteria. Likewise, the addition of repelling nanoparticles into the dispersion of bigger colloidal bacteria particles can also destabilize and flocculate the system by depletion mechanism. Thus, CNCs might be an excellent candidate for creation and manipulation of bacterial flocs and for preventing bacterial initial adhesion and subsequent biofilm development^48,49^. Another study proved the perfect co-localization of CNCs with *E. coli* cells through fluorescent microscopy, supporting the hypothesis that CNCs may directly adhere to them, therefore hindering their adhesion capacities on human intestinal epithelial model cells^50^. Specifically regarding the antimicrobial activity of CNCs on plant pathogenic bacteria, similar results were observed in the work proposed by Schiavi et al.^32^ on *Xanthomonas arboricola* pv. *corylina*, the causal agent of the hazelnut bacterial blight, where similar effects on the in vitro growth inhibition have been recorded for CNCs used at 0.5 and 1% w/v^32^.

All the cited CNCs antibacterial action mechanisms could support the reported results on Psav*. *In vitro growth inhibition values could be explained suggesting the potential role of CNCs as mechanical obstacle to cells’ swimming, since it is one of the most important mechanisms in bacteria’s motility, granting them the ability to quickly colonize liquid environments. We suggest that CNCs could act as a mechanical obstacle to bacterial motility. Also, depletion of bacterial cells could have had a role in inhibit bacterial growth, as well as in lowering Psav biofilm formation. Biofilm formation is a very important capability for bacteria in assuring them survival and colonization of different spaces, phyllosphere included. The interaction of all the aforementioned modes of action could explain the CNCs effect on Psav epiphytic phase. SEM observations of leaves treated with CNC1% at 1 dpi confirmed re-isolation results, revealing a uniform patina enveloping the leaf upper page, hairs included, with few traces of bacterial cells on it, which is the clear sign that CNCs created an unsuitable environment for the adhesion of bacteria and their survival, maybe acting also as mechanical barrier against the colonization of phylloplane.

### Biological interaction of CNCs with olive tree seedlings

In the present study, we also demonstrated the CNCs biocompatibility on olive tree plants by measuring the Nitrogen Balance Index (NBI) and the leaf surface area at different time points. NBI is a non-destructive measure given by chlorophyll and flavonols content ratio in leaves, which can be used as indirect method for estimating the photosynthetic potential of leaves and their nitrogen’s metabolism. When leaves are healthy, most of the nitrogen is involved in the synthesis of chlorophylls; on the contrary flavonols metabolism in response to abiotic and biotic stresses start consuming nitrogen^51^. NBI values were comparable to the controls’ treatments for the duration of the experiments. As expected also leaf area development was not affected by CNCs canopy’s application. For what is in our knowledge, this is the first research study reporting the biocompatibility of CNCs on olive tree plants by observing plant phenotypical parameters. Root uptake in seedling treated with CNCs revealed after 48 h a promising internalization of the nanomaterials, confirmed by confocal and TEM observation, which revealed outside and inside the first layer of root cells the presence of autofluorescent and electrodense materials, absent in controls, attributable for dimension and morphology to CNCs. Mechanisms behind nanomaterials uptake in plants are still largely debated. Nanomaterials on plant surfaces, such as leaves and roots, generally exploit passive modes of entrance. Responses to nanomaterial from root apparatus vary considering the plant species itself (monocot or eudicot) as well as the water availability in culture media. It is believed that the higher root surface area in monocots should make them more sensitive to nanomaterials respect to eudicots, for which nanomaterials uptake could results more difficult. Nevertheless the observed presence of a less compact cortex in plants raised in nutrient solutions could explained also an easier uptake of nanomaterials in comparison with well-drained native soils, in a compatible way of our proposed experiments^52^. The observed CNCs in the first layer of parenchimatic cells of roots suggest a movement from cell to cell which can be delayed by the spontaneous aggregation of nanoparticles in cytoplasm before arriving to the endodermis and vessels. However the internalization of CNCs could open new research lines, highlighting their potential role as sustainable nanocarriers for active ingredients. As far we know this is the first report of root uptake of CNCs in plants, although more studies are needed to deepen the mechanisms behind the ingress of CNCs in root apparatus, as well to investigate their potential translocation in xylem vessels, as already showed for other nanomaterials^52,53^.

This original study underlines the concrete possibility of using CNCs to design sustainable crop protection strategies against bacterial disease, based on agro-industrial wastes valorisation. Cellulose and CNCs could represent a suitable way to reduce copper in agriculture^54^, although their exogenous application has just begun to be studied on model pathosystems. Furthermore very promising results indicate in cellulose another important aspect linked to plant protection, since cellulose oligomers were recognized to act as elicitors of plant defence in *A. thaliana*^55^.

## Methods

### Cellulose extraction and synthesis of cellulose nanocrystals (CNCs)

Olive pruning wastes were collected in Monterotondo, Central Italy, from three local olive cultivars: Carboncella, Frantoio, and Leccino. Shoots with a diameter less than 1 cm were selected for cellulose extraction. After that, olive pruning wastes were accurately washed with deionized water first, then reduced to 3–5 cm and pre-treated in sodium hydroxide (NaOH) at 1% w/v for 72 h and then at 5% w/v for 150 min. After the chemical pre-treatment, the external rhytidome was manually removed and pruning wastes were defibrated by using steel rollers. The resulting biomass was dried at 60 °C for 24 h. Subsequently, the bleaching was performed in sodium chlorite (NaClO_2_) at different concentrations (1, 3, 5% w/v) to investigate the efficacy of such variable on cellulose extraction. The pH of sodium chlorite solutions was adjusted to 3.5 adding acetic acid (CH_3_COOH). The biomass was treated with sodium chlorite at 98 °C for 2 h. The bleached biomass was washed with deionized water and then treated with sodium bisulphate (NaHSO_4_) at 5% w/v for 30 min at room temperature. Following, after being washed with deionized water, obtained fibers were treated with a 17.5% w/v sodium hydroxide solution for 30 min at room temperature. The biomass was washed several times with deionized water and dried at 60 °C for 24 h. The obtained cellulose was hydrolysed in sulphuric acid (H_2_SO_4_) at 64% w/v at 45 °C for 30 min under continuous stirring. The obtained suspension was put in dialysis tubes until pH value of 7 was reached. After that, CNCs aqueous suspension went through an ultrasonic treatment by a dip probe (700 W for 8 min) and stored at 4 °C for next experiments. Acid hydrolysis yield was calculated using the method reported as UNI EN ISO 638:2009^31,41^. Data were obtained from three independent replicates.

### Cellulose nanocrystals characterization

CNCs morphology was investigated by negative staining technique in Transmission Electron Microscopy (TEM). CNCs average dimension was obtained measuring the length and the width of negatively stained crystals, using the software iTEM (Olympus). 120 measurements were taken for each sample. Obtained CNCs were investigated by X Ray Diffraction (XRD) through a PANalytical X’PERT PRO diffractometer source Cu K a k = 1.54184 Å, 40 kV e 40 mA supply, PW3050 goniometer and X’Celerator fast detector using a 0017° 2theta step and 200 s counting time per each step. Crystallinity index (CI) was calculated by following the Segal method: $CI\left(\%\right)=\frac{{I}_{200}-{I}_{am}}{{I}_{200}}\times 100$^40,41,56^.

### Bacterial strain and plant materials for in vitro and in vivo assays

The bacterial strain of *Pseudomonas savastanoi* pv. *savastanoi* (Psav) PvBa206 was furnished by the University of Bari, Italy and firstly isolated in 1968 in Apulia Region from cultivar Ogliarola olive trees^57^. Psav was cultured on King’s B (KB) medium Petri dishes incubated at 27 °C for 48 h.

One year olive tree seedlings cultivar Leccino were purchased from a nursery located in Sammichele di Bari, Italy. Seedlings were propagated by cuttings, 50 cm high, and monocaule. Olive tree seedlings were grown in a glasshouse at 25 ± 2 °C during the day and 16 ± 2 °C during the night with an air relative humidity of 80%.

### In vitro antibacterial assays

In vitro assays were performed to test the inhibitory effect of CNCs against Psav. Four concentrations of CNCs were assayed, 0.05, 0.1, 0.5, and 1% w/v. Moreover, copper sulphate pentahydrate (CuSO_4_∙5H_2_O) (Merck, CAS-number 7758-99-8) was tested as reference compound at the suggested field dose from commercial formulations (0.3% w/v). A 96 microtiter plates assay was conducted using a bacterial suspension containing 1 × 10^4^ CFU/mL, prepared from a fresh culture. CNCs and copper sulphate pentahydrate were suspended in sterile Luria–Bertani broth (LB Broth) at the previously cited concentrations. The obtained solutions were pipetted into the microtiter plates (180 µL) and 20 µL of bacterial suspension was added to each well. The plates were incubated at 27 °C for 48 h. After that the absorbance from the bacterial biomass was measured at OD_600_ by using a DR-200B Microplate reader (Diatek instruments). Negative control (untreated bacterial suspension cultured on LB) was also included. The percentage of growth inhibition was calculated for each tested substance and concentration, after subtracting the blanks (non-inoculated suspensions), by using the following equation:

1 $$\%\, of\, growth\, inibition=100\,\times \frac{Negative \,control-Treated}{Negative\, control}.$$

Data were obtained from three independent experiments, each one consisting of eight replicates^29,58^.

### Bacterial biofilm inhibition assays

To evaluate the effect of CNCs on bacterial biofilm formation, an in vitro assay was made using the following protocol, developed from literature with few adjustments considering the best temperature range for Psav growth^59^. A bacterial suspension (1 × 10^7^ CFU/mL) was obtained from a fresh culture of Psav grown on KB for 48 h at 27°. Different suspensions were made adding CNCs to Tryptone Soy Broth (TSB) (a biofilm-promoting medium) to reach the final concentrations of 0.05, 0.1, 0.5 and 1% w/v. 90 µL of each suspension were poured into the well of a microtiter plate. Then 10 µL of the Psav suspension were added. TSB alone was used as control. Plates were incubated at 27 °C for different time (6, 12 and 24 h). After the incubation plates were washed in deionized water three times and 125 µL of a 0.1% Crystal violet suspension were poured in the wells. Plates were incubated at room temperature for 15 min, then washed in deionized water three time and let dried for 24 h. After that 125 µL of a 30% v/v acetic acid solution were poured in wells and let incubated at room temperature for 15 min. Wells’ content was transferred in a new plate and biofilm quantification was made using a spectrophotometer set at 550 nm (Diatek instruments). Acetic acid alone was used as blank. Data were obtained from three independent experiments, each one consisting of eight replicates.

### In vivo biocompatibility assays

To investigate the effects of CNCs on olive’s foliar development, an evaluation of the CNCs biocompatibility was conducted in a glass house located in Viterbo, Italy (Experimental Farm at University of Tuscia). For this assay, CNCs at 1% were tested since this was the most promising concentration from the previous in vitro experiments. The CNCs suspension was prepared in sterile distilled water as previously described. CNCs, copper sulphate pentahydrate and sterile deionized water, used as controls, were uniformly sprayed on olive tree plants (wetted until runoff). At 1, 7, 14 days post treatment (dpt), the Nitrogen Balance Index (NBI) (DUALEX Scientific + ™) related to the chlorophyll and flavonoid ratio was measured by positioning the instrument in the centre of the leaf shielded from direct sunlight (two measurements per plants)^51^. At the same time, the leaf surface area was measured by using the ImageJ software (version 1.51j8) (NIH, Bethesda, MD, USA) (accessed on Windows 10) (Microsoft, Redmond, WA, USA) (two leaves per plant)^60^. Data were obtained from three independent experiments organized as a complete randomized block, each one consisting of 10 plants for each experimental group.

### In vivo antibacterial assays

The evaluation of the CNCs as antibacterial was conducted in a glass house located in Viterbo, Italy (Experimental Farm at University of Tuscia) to evaluate the potential effects of CNCs on Psav epiphytic survival. The CNCs solution (1% w/v), copper sulphate pentahydrate (0.3% w/v) and sterile deionized water, used as controls were prepared as previously described and uniformly sprayed on olive tree plants (wetted until runoff) 24 h before the Psav artificial inoculation. The Psav inoculum suspension containing 1 × 10^6^ CFU/mL was prepared from fresh cultures on KB medium and homogenously sprayed on olive tree plants using a nebuliser. At 1, 7, 14 days post inoculation (dpi), one treated and inoculated leaf from each olive seedling (ten leaves per thesis) was sampled in a sterile plastic bag and washed with 10 mL of pH 7 potassium phosphate 0.05 M at 110 rpm for 60 s by using a Stomacher^®^ 400 Circulator. Four 1:10 dilutions were performed from the washing water and, 100 µL of each, plated on Petri dishes containing Sucrose Nutrient Agar (SNA) and incubated at 27 °C for 48 h. Psav colonies were morphologically recognized (pale yellow, 1–3 mm diameter, fried egg shape, levan negative) and counted to estimate its epiphytic survival after the treatments. Isolation was repeated thrice for each time point. In order to confirm the Psav identity, colonies were plated on a semi-selective medium composed of sucrose (10 g/L), glycerol (10 mL/L), casamino acids (2.5 g/L), potassium hydrogen phosphate trihydrate (1.96 g/L), magnesium sulphate heptahydrate (0.4 g/L), sodium lauryl sulphate (0.4 g/L), bacteriological agar (16 g/L)^61^. At the same time, the leaf surface area was measured by using the ImageJ software^60^. The number of the Psav counted colonies was related to the leaf surface to determine the CFU/cm^2^. Data were obtained from three independent experiments organized as a complete randomized block, each one consisting of 10 plants for each experimental group^10,29^.

### CNCs uptake in olive plants

To observe the potential uptake of CNCs by olive root apparatus, a 1% w/v CNCs suspension was prepared using an Hoagland solution as liquid medium. Three olive seedlings were removed from pots, accurately washed with deionized water, and put in the suspension under continuous stirring. Hoagland solution alone was used as control. After 48 h roots were cut to be prepared for confocal and TEM observation.

### Sample preparation for confocal observation

Longitudinal and cross section (20 µm) of primary roots were obtained by a cryostat. Briefly, samples were placed on a tissue holder, covered with an embedding medium (OCT), and then immersed into liquid nitrogen. Sections were mounted onto positively charged slides and stored at − 20 °C until use. Following washes in phosphate buffer 0.1 M, sections were coverslipped in an aqueous mounting medium (Fluoroshield, Merk).

### Confocal microscope observation

Slides were analysed with a confocal microscope system (Zeiss LSM 710) and images were acquired using the interfaced software ZEN 2010: both microscope hardware and software configuration were always maintained. Technical parameters fixed in our acquisition procedure were pinhole size, at 1 AU (Airy unit), laser power at 2%, and digital gain at 1.0. Images were then processed using ImageJ software, to merge channels from monochrome acquisitions and make montage, when serial microscope scans of the specimen were performed along z axis.

### Electron microscopy

For Negative staining of CNCs, droplets of sample suspensions (10 µL) were placed on formvar-carbon coated grids and allowed to adsorb for 60 s. Excess liquid was removed gently touching the filter paper. The adsorbed specimen was then processed for negative-staining, by first washing the specimen grid on a drop of negative stain (2% uranyl acetate in distilled water), blotting and repeating this step once more, this time leaving the specimen grid for 60 s on a new drop of negative stain solution. Samples were observed at a JEOL 1200 EX II electron microscope. Micrographs were acquired by the Olympus SIS VELETA CCD camera equipped the iTEM software. For Scanning Electron Microscopy (SEM) analyses, leaves of olive tree were fixed using osmium vapors, to avoid that any material deposited on the surface of the leaf could be washed off by immersing the sample in a fixative solution. The subsequent preparation process was done without ever immersing the sample in a solution. Samples were dried in the air, then they were attached to aluminium stubs using a carbon tape and sputter-coated with gold in a Balzers MED 010 unit. The observations were made by a JEOL JSM 6010LA electron microscope. For Transmission Electron Microscopy (TEM), small samples were removed for the entire olive roots using a razon blade and fixed overnight at 4 °C with 2.5% (v/v) glutaraldehyde + 2% (v/v) paraformaldehyde in 0.1 M cacodylate buffer, pH 7.2. After 3 × 20 min washings at 4 °C in the same buffer, samples were post-fixed with 1% (v/v) osmium tetroxide in 0.1 M cacodylate buffer, pH 7.2 for 2 h at 4 °C. Specimens were washed in the same buffer (3 changes for 15 min each at 4 °C), and then dehydrated in a graded ethanol series, followed by 2 changes for 10 min at 4 °C in propylene oxide. Samples were then infiltrated with mixtures of Agar 100 resin/propylene oxide in different percentages. At the end of the procedure, samples were embedded in pure Agar 100 resin, and let to polymerize for 2 days at 60 °C. Resin blocks were cut with Reichert Ultracut ultramicrotome using a diamond knife. Ultrathin section (60–80 nm) were collected on copper grids, stained with uranyl acetate and lead citrate, and observed with a JEOL 1200 EXII electron microscope. Micrographs were captured by the Olympus SIS VELETA CCD camera equipped with iTEM software.

### Statistical analysis

Data from in vitro experiments, in vivo biocompatibility, and antibacterial experiments were subjected to one-way analysis of variance (ANOVA). Two level of significance (*p* < 0.05 and *p* < 0.01) were computed to assess the significance of the F values. A pairwise analysis was carried out using the Fisher’s least significant difference (LSD) test at 0.95 or 0.99 confidence level. Statistical analyses were performed using XLSTAT 2020.4 (Addinsoft, France).
