# Author Correction: Exploring cellulose nanocrystals obtained from olive tree wastes as sustainable crop protection tool against bacterial diseases

**DOI:** 10.1038/s41598-022-11195-8

**Published:** 2022-04-25

**Authors:** Daniele Schiavi, Sara Francesconi, Anna Rita Taddei, Elena Fortunati, Giorgio M. Balestra

**Affiliations:** 1 Department of Agriculture and Forest Sciences (DAFNE), University of Tuscia, Via S. Camillo de Lellis Snc, 01100 Viterbo, Italy; 2 High Equipment Centre, Section of Electron Microscopy, University of Tuscia, Largo dell’Università Snc, Blocco D, 01100 Viterbo, Italy

Correction to: *Scientific Reports* 10.1038/s41598-022-10225-9, published online 12 April 2022

The Acknowledgements section in the original version of this Article was incomplete.

“Authors gratefully thank MUR (Italian Ministry for University and Research) (Law 232/2016, Departments of Excellence); Proff. Nicola S. Iacobellis and Giuseppe Surico for provided Psav (PvBa206) bacterial strain; Prof. Davide Cervia, Dr. Elisabetta Catalani, Dr. Elena Paccosi for the help given in preparing and observing samples by confocal microscope.”

now reads:

“Authors gratefully thank MUR (Italian Ministry for University and Research) (Law 232/2016, Departments of Excellence) and PON Ricerca e Innovazione 2014-2020. Nanotecnologie chimiche green per la protezione sostenibile delle piante (NEMESI) ARS01_01002 CUP: F36C1800018000X); Proff. Nicola S. Iacobellis and Giuseppe Surico for provided Psav (PvBa206) bacterial strain; Prof. Davide Cervia, Dr. Elisabetta Catalani, Dr. Elena Paccosi for the help given in preparing and observing samples by confocal microscope.”

The original Article has been corrected.

